# Multi-omics analyses revealed three Golgi apparatus genes potentially associated with poor prognosis in colorectal cancer patients

**DOI:** 10.1590/1678-4685-GMB-2024-0126

**Published:** 2025-09-26

**Authors:** Peng Zhu, Shisi Shen, Xi Wang, Jie Li, Donge Tang, Yong Dai, Min Tang, Wei Zhang, Guoping Sun

**Affiliations:** 1 Southern Medical University, Pingshan Hospital, Shenzhen Pingshan District People’s Hospital, Shenzhen, China.; 2 Shenzhen University, South China Hospital, Health Science Center, Shenzhen, China.; 3 Chinese Academy of Medical Sciences and Peking Union Medical College, Peking Union Medical College Hospital, Departments of Obstetrics and Gynecology, Beijing, China.; 4 University of Chinese Medicine, Shenzhen Traditional Chinese Medicine Hospital, The Fourth Clinical Medical College of Guangzhou, Shenzhen, China.; 5 Peking University Shenzhen Hospital, Department of Clinical Laboratory, Shenzhen, China.; 6 Anhui University of Science and Technology, School of Medicine, The First Affiliated Hospital, Huainan, China.; 7 Chongqing Medical University, Key Laboratory of Laboratory Medical Diagnostics designated by Chinese Ministry of Education, Chongqing, China.

**Keywords:** Colorectal cancer, prognostic biomarkers, Golgi apparatus, multi-omics study, transcription factors

## Abstract

The identification of novel functional biomarkers is crucial in recognizing high-risk colorectal cancer (CRC) patients. Despite this need, no prognostic biomarker has been implemented in clinical practice for CRC. To address this gap, we utilized integrated transcriptomic data from public databases alongside our original multi-omics data, including proteome and chromatin accessibility datasets. Bioinformatics studies on transcriptomic datasets from 487 CRC patients led us to identify three Golgi apparatus prognostic genes: NIPAL1, ZYG11B, and PARP10. We found that decreased expression of NIPAL1 and ZYG11B, as well as increased expression of PARP10, elevated the risk of CRC. These genes are potentially involved in cellular processes such as nucleotide excision repair and DNA replication. Additionally, our original multi-omics datasets, encompassing proteomic data and chromatin accessibility profiling from assay for transposase-accessible chromatin with sequencing (ATAC-Seq), identified alterations in protein levels of potential upstream transcription factors CDX2 and YY1 for three genes. Furthermore, chromatin accessibility at DNA binding regions corresponding to transcription factors such as SPI1 and JUND changed, potentially explaining the observed variations in mRNA levels for these genes. Our findings highlight the biological activities of these genes, including NIPAL1, PARP10, and ZYG11B, and their upstream regulators, offering a functional context for future in-depth mechanistic studies.

## Introduction

Colorectal cancer (CRC) ranks the third most common cancer in the world in the 2020 Global Cancer Statistics (Sung et al., 2021; Zhao et al., 2022). It is estimated that in 2020 there were more than 9.1 million new diagnoses and 935,000 deaths of CRC, ranking the second highest CRC mortality among all cancers (Sung *et al.,* 2021). In addition, more than 20% of CRC patients are already in advanced stages of cancer at diagnosis (Song et al., 2022). Therefore, if we are able to identify significant potential biomarkers, we can enhance the rate of CRC detection. It is crucial to initiate clinical therapy at the earliest possible stage, and effectively reduce CRC mortality.

According to research, the tissues of CRC generate and release abnormally glycosylated proteins (Kellokumpu, 1986; Rhodes et al., 1986; Kellokumpu *et al.,* 2002). These proteins, which have abnormal glycosylation, may play a role in biological processes such as intracellular protein sorting and signal transmission (Paulson, 1989). It is believed that these abnormally glycosylated proteins could be important for cancer invasion and metastasis (Kellokumpu, 1986). The Golgi apparatus playing a significant role in post-translational modification and sorting of proteins, is responsible for transporting proteins to vesicles for delivery to specific target sites (Millarte and Farhan, 2012). In recent years, several Golgi genes have been identified as potential predictive indicators for CRC. One such gene is GS28, which is involved in ER-Golgi transportation (Subramaniam et al., 1996). A retrospective analysis was conducted to study the prognostic usefulness of GS28 in CRC patients. The study revealed that nuclear predominant expression of GS28 can serve as a prognostic marker for CRC and aid in identifying aggressive forms of the disease (Lee et al., 2017). However, many studies suffer from small sample sizes, and the use of biomarkers in CRC clinical practice is often limited (Lee *et al.,* 2017; Echeverri and Orozco, 2022). Therefore, it is crucial for researchers to identify more significant biomarkers that can be utilized by clinical professionals.

In this study, we conducted a survival analysis on RNA-Seq datasets of a total of 51 tumor-adjacent tissue samples and 487 tumor samples, and identified 913 genes associated with survival. 168 genes were subjected to Gene Ontology (GO) enrichment analysis, with 52 being protective genes with modest expression and 116 being hazardous genes with elevated expression. We performed GO enrichment analysis on these genes and found that they were mainly enriched in the Golgi apparatus, highlighting the strong connection between the Golgi apparatus and CRC. We further investigated three specific genes that were linked to both the Golgi apparatus and CRC survival. Co-expression enrichment analysis was utilized to identify the possible biological functions of these three genes. In addition, we explored the potential upstream transcription factor changes in the multi-molecular dimension of these three genes using our own raw data including proteome data and the assay for transposase-accessible chromatin with sequencing (ATAC-Seq) data. These findings provide potential novel targets and methods for CRC treatment.

## Methods

### Data Repository and Access

Mass spectrometry proteomics data were unloaded and deposited into the ProteomeXchange Consortium through the PRIDE partner repository as entries designated PXD021314. Data of ATAC-Seq were unloaded and deposited into the Sequence Read Archive (SRA) with accession to PRJNA693028 (Zhang et al., 2021).

### Human Protein Atlas (HPA)

Based on the immunohistochemical method, the HPA database provides the protein levels of NIPAL1, PARP10, and ZYG11B in CRC tissue and normal colorectal epithelial tissue samples.

### Linkedomics

The Linkedomics database is a comprehensive multi-omics database that integrates miRNA data, mRNA data, methylation data, mutation site data and clinical data for 32 different cancer types and 11,158 patients (Vasaikar et al., 2018). In this study, we utilized the LinkFinder module to generate heatmaps depicting the top 50 proteins that are positively and negatively correlated with three specific genes (NIPAL1, PARP10, and ZYG11B).

### CancerSEA

CancerSEA aims to comprehensively investigate the cellular-level biological processes that cancer cells are involved in, such as Epithelial-Mesenchymal Transition (EMT), proliferation, apoptosis, DNA repair, and other processes (Yuan et al., 2019). The database provides insights into the biological functional profiles of 41,900 individual cells derived from 25 distinct types of human cancers (Yuan *et al.,* 2019). In this study, we utilized cancerSEA to explore the underlying biological mechanisms associated with the three genes (NIPAL1, PARP10, and ZYG11B).

### TFtarget

TFtarget is currently the most comprehensive and extensive database available that documents the relationship between human transcription factors and their targets (Stewart et al., 2018). TFtarget integrates high-confidence DNA-binding sequences from 699 transcription factors, along with 2737 TFBS motifs from these 699 TFs. We obtained the experimental confirmation of each transcription relation with the genes (NIPAL1, PARP10, and ZYG11B) from TFtarget, establishing their connection.

### RNA-Seq data processing and differential expression analysis

The RNA-Seq datasets of 638 CRC and 51 tumor-adjacent tissue samples were obtained from the TCGA. After excluding samples with gene expression deletions and incomplete survival times, a total of 51 tumor-adjacent tissue samples and 487 tumor samples were included in the analysis. For preliminary quality control, low-quality measurements and joint sequences were eliminated using the FastQC program. In order to remove variations in sequencing depth and gene length between samples and guarantee the comparability of expression data, the quantity of gene expression was normalized and the FPKM (fragments per kilobase of exon per million reads mapped) approach was used. Lastly, differential gene analysis was carried out on normalized data using “DESeq2” R package for differential expression analysis. P-value was corrected by FDR (False Discovery Rate), and genes with FDR < 0.05 and fold change >2 were selected out as differential genes.

### Cox analysis of gene expression and CRC risk

Using clinical data from CRC patients in the TCGA database, we extracted patients’ survival time (from surgery to death or last follow-up) and survival status (death = 1, survival = 0). Gene expression levels (after FPKM normalization) were selected for analysis. All gene expression data were preprocessed to ensure standardization and normalized with FPKM, controlling for differences in sequencing depth and gene length across samples. Genes with missing values in more than 40% of samples were excluded. Based on the median expression level, genes were categorized into high and low expression groups. The ‘survival’ R package was employed for univariate Cox regression analysis to calculate the hazard ratio (HR) for each gene, assessing the impact on patient survival. An HR > 1 indicates that increased gene expression elevates the risk of CRC, while an HR < 1 suggests that higher gene expression reduces the risk of CRC progression.

### Pearson correlation and statistical significance

The Pearson correlation coefficient was employed to evaluate the association between the expression levels of genes. *P*-values less than or equal to 0.05 were considered statistically significant. The symbols ‘*’, ‘**’, and ‘***’ were used to indicate *p*-value less than 0.05, 0.01, and 0.001, respectively. The statistical analysis and visualization were performed using R version 4.0.3.

## Results

### The transcriptomic analysis revealed three Golgi apparatus genes as potential prognostic biomarkers for CRC

**Figure 1 -:**
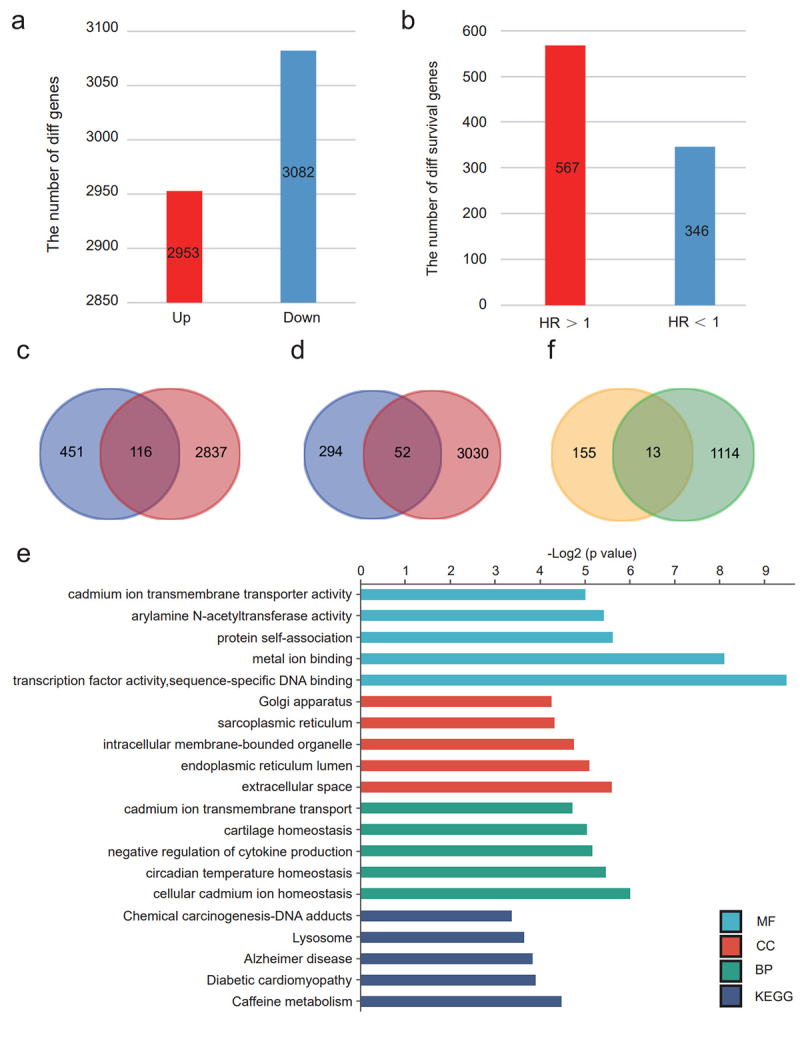
Three Golgi apparatus genes were identified as potential predictive biomarkers for human CRC through transcriptomic analyses. a)The number of genes in CRC demonstrating differential expression. b)The number of differentially expressed survival genes in CRC. c) The intersection of 567 risk factor genes and 2953 highly expressed genes. d) The intersection of 346 protective genes and 3082 lowly expressed genes. e) The Gene Ontology (GO) enrichment of the 168 genes. f) The intersection of 168 genes and 1,127 Golgi-associated genes.

To investigate potential risk-predictive markers for CRC, we first screened differentially expressed genes in 487 CRC tissue versus 51 para-cancer tissue samples from TCGA. Our analysis identified 2,953 genes with high expression and 3,082 genes with low expression in CRC tissue versus para-carcinoma tissues (Figure 1 a ). Next, we conducted a univariate Cox regression analysis on the dataset to identify risk-associated genes. As a result, we quantified 567 genes with hazard ratios (HR) greater than 1 and 346 genes with HR less than 1 (Figure 1 b). To ensure alignment between gene expression levels and HR values-linking high-expression genes to poor prognosis and low-expression genes to better prognosis-we intersected the 2,953 highly expressed genes with the 567 risk factor genes, resulting in 116 genes. Similarly, intersecting the 3,082 low-expression genes with the 346 protective genes yielded 52 genes (Figure 1 c-d ). Combining these two sets, we identified a total of 168 genes as CRC risk-associated genes, which are likely involved in the initiation and progression of CRC.

To elucidate the biological processes primarily involving these 168 genes, we performed a GO enrichment analysis. The results indicated that these genes were primarily enriched in the Golgi apparatus, as well as in the sarcoplasmic reticulum, intracellular membrane-bounded organelles, and the endoplasmic reticulum lumen (Figure 1 e ). This result elucidated that the prognosis of individuals with CRC may be influenced by the dysfunctions of Golgi apparatus. Meanwhile, the Golgi has a central role in protein processing and transport, which is essential for maintaining cellular function. Although the basic functions of the Golgi apparatus are well known, little is known about the specific molecular roles and regulatory mechanisms of the Golgi apparatus in pathological processes such as cancer. Many studies have focused on other organelles, such as the endoplasmic reticulum and mitochondria (Zong et al., 2016; Chen and Cubillos-Ruiz, 2021), but alterations in the Golgi may affect secretion and signaling pathways in cancer cells, which may have important implications for tumor progression (Choi et al., 2022). Consequently, we focused on the investigation of Golgi genes in our work.

To clarify which Golgi apparatus genes are involved in the development of CRC, we extracted Golgi-related genes from the set of 168 risk-associated genes. We first identified 1,127 Golgi proteins from the HPA database. By overlapping these 1,127 Golgi-associated genes with the 168 risk genes, we identified 13 genes that were not only Golgi-related but also correlated with the survival prognosis of CRC patients (Figure 1 f ). Among these 13 genes, we noticed that the functions of NIPAL1, PARP10, and ZYG11B are closely associated with cancer. NIPAL1 may play an important role in the transport and regulation of calcium and magnesium plasma (Goytain et al., 2008; Manialawy et al., 2020), calcium ions play a regulatory role in the proliferation, apoptosis and migration of CRC cells (Yang et al., 2019). NIPAL1, as a gene regulating ion homeostasis, may play a key role in the development of CRC. PARP10 is an ADP-ribosylase involved in DNA repair and maintenance of genomic stability (Szanto et al., 2021; Khatib et al., 2024). The PARP family plays a key role in DNA damage response, regulating cellular repair and apoptosis responses to DNA damage (Szanto *et al.,* 2021; Khatib *et al.,* 2024). Dysregulation of DNA damage repair pathway is one of the key features of CRC. The activity of PARP10 may affect the sensitivity and repair ability of CRC cells to DNA damage, thus affecting their proliferation and survival. In addition, members of the PARP family have been validated as drug targets in cancer therapy (Szanto *et al.,* 2021), and PARP10 has the potential to be a therapeutic target for CRC. Uncontrolled cell cycle is a core feature of cancer development, and ZYG11B, as a regulator of cell cycle, may play a role in the regulation of CRC cell proliferation and apoptosis (Zhou et al., 2024). In addition, we found that the role of these three Golgi genes in CRC has not been studied in a large cohort, so we selected three genes, NIPAL1, PARP10, and ZYG11B, for the subsequent studies.

**Figure 2 -:**
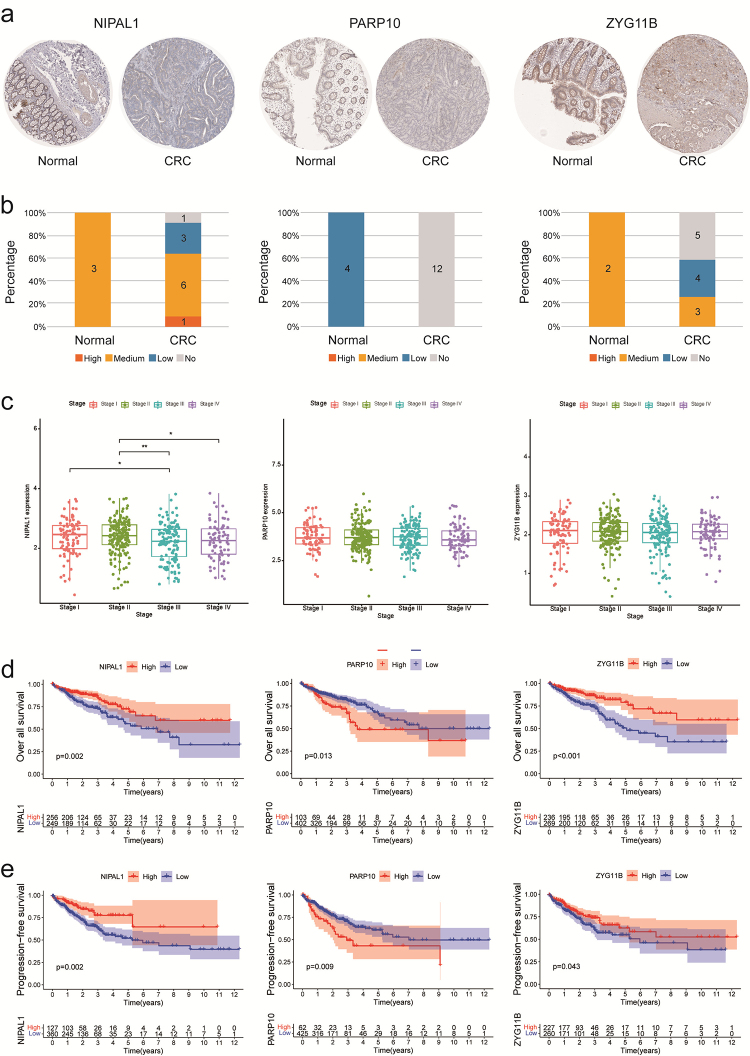
NIPAL1, PARP10, and ZYG11B were associated with the survival of CRC patients. a-b) The immunohistochemical measurement of NIPAL1 (normal = 3, tumor = 11), PARP10 (normal = 4, tumor = 12) and ZYG11B (normal = 2, tumor = 12) in normal and colon adenocarcinoma tissues obtained from The Human Protein Atlas and statistical results. c) The relationship between mRNA expression and disease progression. d) The relationship between mRNA expression and overall survival rates of the patients (N=505). e) The relationship between mRNA expression and progression-free survival rates of the patients (N=487). * *p* value < 0.05, ** *p* value < 0.01.

To investigate the role of NIPAL1, PARP10, and ZYG11B in CRC tumorigenesis, we further analyzed the protein expression of these three genes in both CRC tissues and normal colorectal epithelial tissues obtained from the HPA database (Figure 2 a-b ). Our findings revealed that a majority of CRC tumors showed significantly decreased levels of NIPAL1 and ZYG11B expression versus normal tissues, while PARP10 expression was undetectable in CRC tumors compared to normal tissues. 

Subsequently, we investigated the expression of these three genes at various stages of cancer progression. Our findings revealed that NIPAL1 expression consistently declined as the disease progresses, suggesting a potential involvement of NIPAL1 in cancer progression, including invasion and metastasis. Conversely, the expression levels of PARP10 and ZYG11B remained relatively stable throughout the course of the tumor (Figure 2 c ). We classified the expression of genes into high expression group and low expression group based on the optimal cut-off points to assess the survival analysis of three genes. It was observed that higher expression of PARP10 in CRC tissues was associated with shorter overall survival (OS) of patients, while lower expression of NIPAL1 and ZYG11B was linked to shorter OS (Figure 2 d ). Furthermore, the analysis of patients’ progression-free survival (PFS) and the expression of the three genes revealed that shorter PFS was associated with lower expression of NIPAL1 and ZYG11B and higher expression of PARP10 (Figure 2 e ).

**Figure 3 -:**
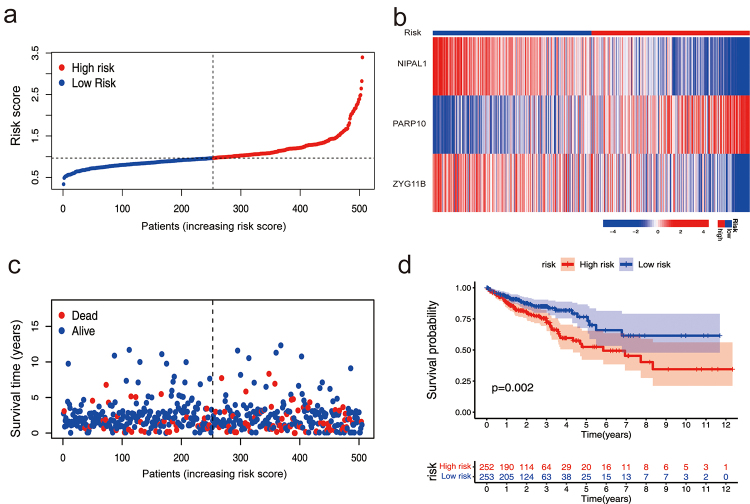
NIPAL1, PARP10, and ZYG11B acted as a signature could differentiate CRC patients at high risk from low risk. a-c) CRC patients were divided into high-risk and low-risk groups based on the expression of these three genes. d) The overall survival rate of patients in high-risk and low-risk groups.

CRC patients were classified into high-risk and low-risk groups based on a risk score calculated from the median values of three genes (Figure 3 a-c ). The heat map displayed the different risk groups. The high-risk group was characterized by low expression of NIPAL1 and ZYG11B, and high expression of PARP10. Conversely, the low-risk group was characterized by high expression of NIPAL1 and ZYG11B, and low expression of PARP10. The survival curve demonstrated a significant difference between the high-risk and low-risk groups, indicating that the high-risk group had a shorter survival period (*p*=0.002) (Figure 3 d ).

### Co-expression analyses demonstrated that the three genes potentially collaborate in nucleotide excision repair and DNA replication

**Figure 4 -:**
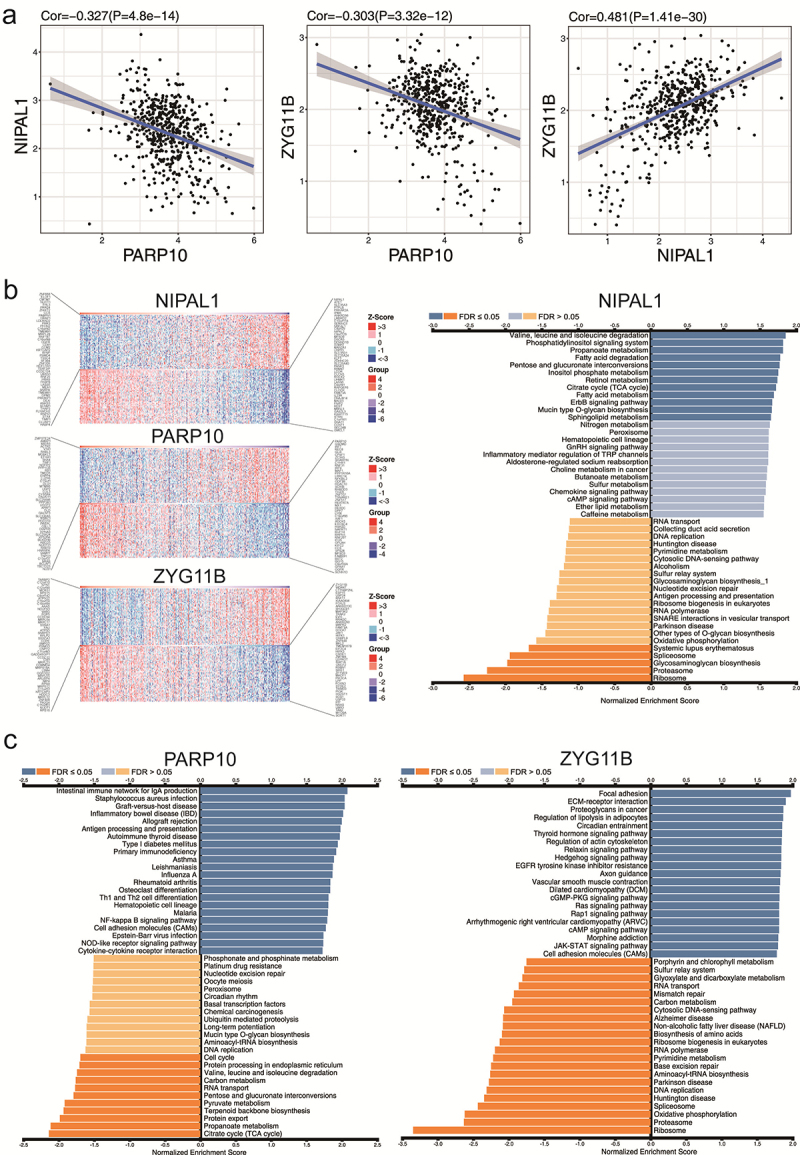
Potential biological functions of NIPAL1, PARP10 and ZYG11B. a) The co-expression interactions of these three genes using RNA-Seq datasets obtained from TCGA. b) Heat map of 50 genes which are either negatively or positively associated with NIPAL1, PARP 10 and ZYG11B. c) The results of KEGG enrichment analysis, where the top 25 pathways demonstrate a positive correlation with NIPAL1, PARP10, and ZYG11B, whereas the last 25 pathways exhibit a negative correlation with these genes.

The functions of NIPAL1, ZYG11B, and PARP10 in the occurrence and progression of CRC were previously unclear. In this study, we investigated the co-expression interactions of these three genes using RNA-Seq datasets obtained from TCGA. Our findings revealed a significant co-expression relationship between NIPAL1, PARP10, and ZYG11B (co-expression coefficient > 0.3 or < -0.3) (Figure 4 a ), suggesting their potential involvement in shared biological processes. It is observed that the mRNA expression of NIPAL1 and ZYG11B may be co-expressed in CRC, while the mRNA expression of PARP10 shows an opposite trend. Additionally, we identified the proteins co-expressed with these three genes using the Linkomics database (Figure 4 b ). KEGG enrichment analysis further indicated that the functions of NIPAL1, ZYG11B, and PARP10 were potentially related to nucleotide excision repair and DNA replication, highlighting their potential involvement in these two processes (Figure 4 c ). On the other hand, the functions of NIPAL1 were found to be primarily involved in degradation of amino acids, such as valine, leucine, and isoleucine degradation. Additionally, ZYG11B displayed a favorable correlation with several crucial cancer-relevant pathways, including the Ras signaling system, the Ras-cGMP-PKG pathway, and the regulation of lipolysis in adipocytes. PARP10 was found to be relevant to the NF-kappa B pathway and cytokine-cytokine receptor interaction, both of which are important pathways in immune response regulation. Our KEGG analysis was performed based on the co-expressed genes of the target genes, and the results showed that these genes are involved in many biological processes that are not directly related to the Golgi. This reflects the complexity and interconnectivity of cellular functions such that genes associated with the Golgi apparatus may also be involved in a broader biological network. These findings suggest that the Golgi apparatus, although primarily known for protein processing, may indirectly influence other pathways through its network of molecular interactions and co-expressed genes. 

**Figure 5 -:**
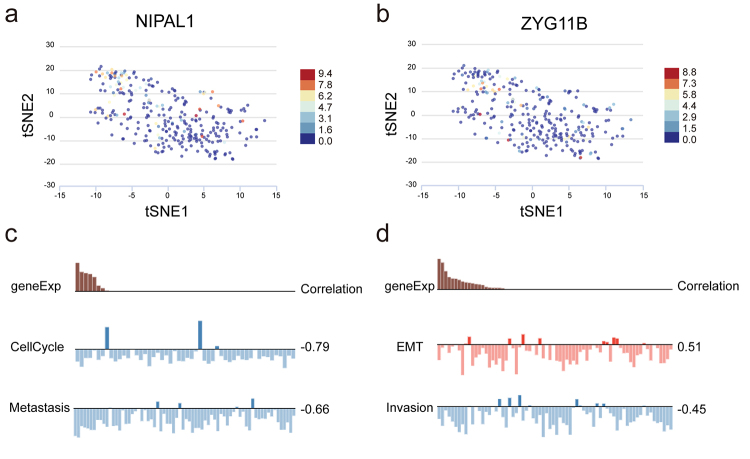
NIPAL1 and ZYG11B were potentially associated with a series of tumor-related biological processes including cell cycle, invasion, metastasis. a-b) The differential expression of NIPAL1 and ZYG11B in single CRC cells. c-d) NIPAL1 and ZYG11B were potentially related to multi-functions of cells.

Further investigation was conducted to examine the potential biological functions of these three genes in CRC using single-cell sequencing datasets from the CancerSEA database. The results revealed distinct expression patterns of NIPAL1 and ZYG11B in various CRC cells (Figure 5 a-b). Consistent with our previous findings from bulk RNA-Seq analysis, NIPAL1 and ZYG11B were found to be down-regulated in CRC tissues compared to normal adjacent tissues. However, the single-cell sequencing results provided additional insights. Specifically, NIPAL1 was found to be adversely associated with metastasis and cell cycle (Figure 5 c), while ZYG11B showed a positive correlation with EMT and a negative correlation with invasion (Figure 5 d ). These findings suggest that the presence of NIPAL1 and ZYG11B may have a preventive effect on tumor progression, including invasion and metastasis. Furthermore, ZYG11B may exhibit dual roles in CRC tumorigenesis, promoting the transformation of normal cells into tumor cells in the early stages and inhibiting tumor progression in the late stages.

### Deciphering the potential upstream transcription factors of the three genes

**Figure 6 -:**
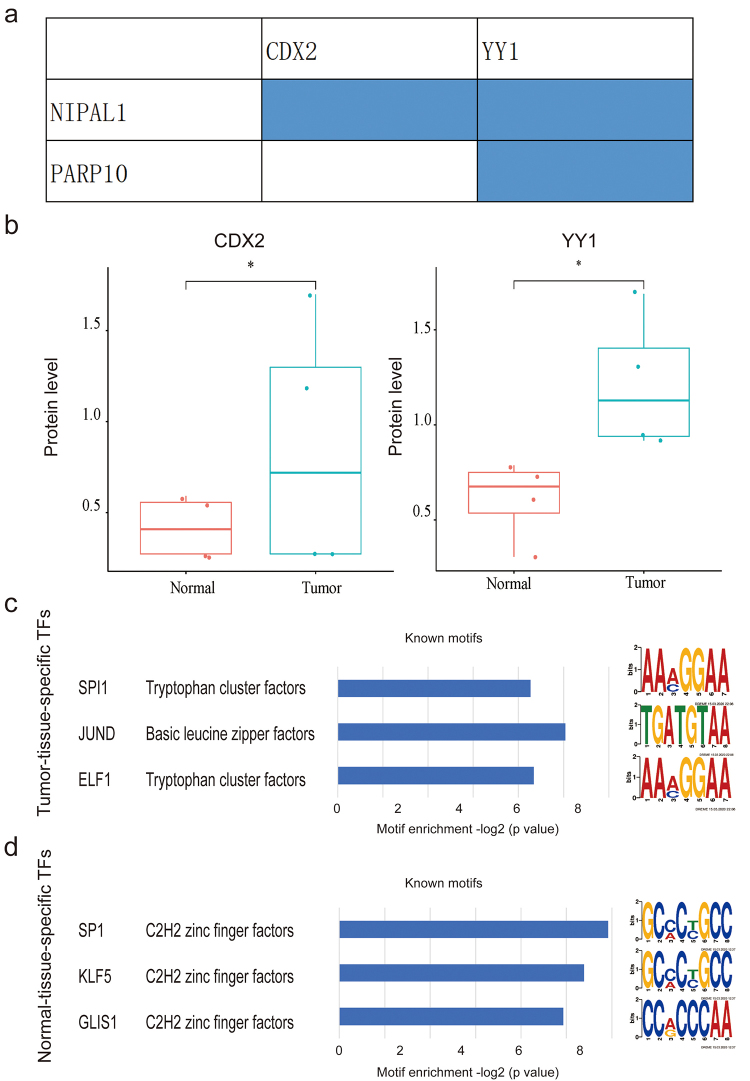
Comprehensive investigations unveiled altered amount of protein level or binding motifs on the three golgi apparatus genes’ potential transcriptional factors. a) Blue represents the potential regulatory relationship between transcription factors CDX2 and YY1 on NIPAL1 and PARP10 in CRC tissues. b) Protein expression of CDX2 and YY1 in CRC and para-cancer tissue samples. c) The accessibility of DNA binding motifs of the potential transcriptional factors in CRC tissues. d) The accessibility of DNA binding motifs of the potential transcriptional factors in para-cancer tissues. * *p* value < 0.05

To explore the reasons for the aberrant expression of PARP10, NIPAL1 and ZYG11B in CRC tissue samples versus normal samples, we analyzed their potential upstream transcription factors from different dimensions, including DNA methylation, structural variation, chromatin accessibility, protein level and phosphorylation sites. Firstly, we identified their experimentally validated upstream transcription factors in colorectal tissues based on the TFtarget database. Next, we examined the alterations of these transcription factors across different molecular dimensions. As a result, we observed changes in protein levels for CDX2 and YY1 as transcription factors of NIPAL1, and YY1 as the transcription factor of PARP10 (Figure 6 a-b). Furthermore, we analyzed our original chromatin accessibility data, and found that the DNA binding motifs of ELF1, JUND, and SPI1, which are potential transcription factors of PARP10, NIPAL1 and ZYG11B in CRC tissues, are specifically open in cancer tissues compared with adjacent tissues. However, GLIS1, KLF5, and SP1 were only found in the adjacent tissues (Figure 6 c-d ). These findings suggest that the differential expression of the three genes may be attributed to changes in the protein levels of transcription factors and changes in the accessibility of the corresponding DNA-binding regions.

## Discussion

The prognosis for CRC, the third most prevalent cancer worldwide, remains unsatisfactory (Zhao et al., 2022). In this study, we discovered that the Golgi apparatus is enriched in a substantial number of genes related to CRC survival. We specifically confirmed the differential expression of three Golgi-related genes (NIPAL1, PARP 10 and ZYG11B) between tumor and para-cancer tissue. Additionally, we examined the association between the expression of each gene and patients’ OS and PFS. Using these three genes as features, we were able to distinguish high-risk groups from low-risk groups and predict CRC patient survival. We further explored the potential roles of these genes in the molecular mechanisms underlying cancer, particularly in nucleotide excision repair and DNA replication, as evidenced by enrichment studies of co-expressed proteins. Furthermore, our findings suggest that NIPAL1 and ZYG11B may have a preventive effect on carcinogenesis and growth, as indicated by cancerSEA. Lastly, we investigated the reasons for the aberrant expression of three genes in CRC versus normal colorectal epithelium samples.

At the time of initial diagnosis, 20% of patients already have advanced malignancy, with liver metastasis being the most common site of metastasis (Song et al., 2022). The 5-year survival rate for all patients with metastatic CRC is less than 20% (Stewart et al., 2018). These findings highlight the urgent need for prognostic biomarkers to aid in clinical diagnosis and treatment of CRC. The workload of healthcare professionals could be significantly reduced by using credible biomarkers to classify high- and low-risk CRC patients. This would simplify the process of following up on patients, performing effective clinical interventions, and making timely changes to treatment plans. However, no unique biomarker genes have been identified for the clinical diagnosis of CRC. This article has discovered three Golgi-related genes-NIPAL1, ZYG11B, and PARP10-which may provide new insights into identifying and treating CRC.

The link between the Golgi apparatus and tumor invasion and metastasis has been extensively researched, with a particular emphasis on its involvement in CRC. Experimental studies have revealed that Golgi transport 1B (GOLT1B) encodes the vesicle transporter of the Golgi apparatus (Liu et al., 2021). In CRC, a high expression of GOLT1B leads to increased levels of DVL2 and enhances plasma membrane translocation. This activation of the downstream Wnt/β-catenin pathway further promotes the migration and invasion of cancer cells (Zhu et al., 2018). These findings support the notion that the Golgi apparatus could be a potential target for CRC treatment. By focusing on the connection between the Golgi apparatus and CRC, we identified three Golgi-related genes (NIPAL1, PARP 10 and ZYG11B), and found that the expression of these three genes were associated with patient OS and PFS. These findings further support the notion that these three Golgi apparatus genes could potentially serve as functional genes for CRC.

NIPAL1 is responsible for magnesium ion transport and encodes proteins (Zhu et al., 2018). PARP10, a member of the mono-ADP ribosyltransferase family, regulates gene transcription and DNA damage repair (Kaufmann et al., 2015). Additionally, ZYG11B is involved in proteasome ubiquitin-dependent catabolic processes as part of an E3 ubiquitin ligase complex (Timms et al., 2019). Our findings indicate that these three genes may participate in a shared biological process as positive or negative regulators, with enrichment analysis of their co-expressed proteins highlighting roles in nucleotide excision repair and DNA replication. 

There is a growing body of evidence linking nucleotide excision repair with tumorigenesis. Xeroderma pigmentosum (XP), a condition characterized by impaired DNA repair, leads to pigmentation in sun-exposed skin areas and significantly increases the risk of skin cancer (Fayyad et al., 2020). In a study conducted by Sylwia Pietrasik, the hypothesis was proposed that the interaction between BRCA1 and GADD45 can lead to the over-repair of DNA damage, thereby increasing the risk of breast cancer (Pietrasik *et al.,* 2020). Another study (Domingo et al., 2016) focused on CRC and found that mismatch repair deficient (MMR-D) CRC showed an enhanced immune response and a reduced risk of recurrence compared to mismatch repair proficient (MMR-P) CRC. This suggests that nucleotide excision repair plays a crucial role in the development of CRC (Domingo *et al.,* 2016). 

Additionally, in our investigation into the potential biological significance of ZYG11B in CRC, we discovered an interesting phenomenon. We observed that the expression of ZYG11B decreased as CRC progressed, while it was positively associated with EMT and inversely correlated with invasion. Based on these findings, we proposed that ZYG11B may have a dual role in the development of CRC. Specifically, it may promote tumor progression in the early stages and inhibit invasion of the tumor later on. Previous studies have suggested that certain molecular pathways or transcription factors can have dual roles in tumor development. For example, Nur77, a member of the orphan receptors family, has been reported to play a dual role in the progression of several cancers, including CRC (Wilson et al., 2003), hepatocellular carcinoma (Bian et al., 2017), and gastric cancer (Wu et al., 2002). The underlying mechanism of this dual relationship in Nur77 has also been extensively studied. Therefore, building on these previous studies, our study proposes the hypothesis that ZYG11B may have a dual regulatory role in the progression of CRC. Further research is needed to investigate this hypothesis. To sum up, our study is innovative and raises many possibilities and ideas, especially ZYG11B. 

The study has certain limitations. Firstly, although the protein expression of these three genes has been verified by tissue samples in the HPA database, the number of samples is small (n_normal_=2-4, n_cancer_=11-12), so we still need to verify it in a larger cohort. Secondly, this study identified NIPAL1, PARP10, and ZYG11B as potential prognostic biomarkers for CRC through multi-omics analysis, but further experimental validation is needed to elucidate the specific functional roles of these genes in CRC progression. Future studies with functional validation including *in vivo* and *in vitro* experiments will be key to confirm and extend our findings, especially in understanding how these genes influence tumorigenesis and patient prognosis. Nevertheless, our study provides a basic framework for subsequent studies, suggesting that these genes are potential targets for CRC research.

## Conclusions 

In conclusion, the present findings confirm that NIPAL1, PARP10, and ZYG11B can serve as biomarkers for the prognosis of CRC. These three genes may be involved in the biological processes of nucleotide excision repair and DNA replication, offering new avenues for the future treatment and diagnosis of CRC.

## Data Availability

 Mass spectrometry proteomics data has been uploaded and deposited into the ProteomeXchange Consortium through the PRIDE partner repository as entries designated PXD021314. Data of ATAC-Seq has been uploaded and deposited into the Sequence Read Archive (SRA) with accession to PRJNA693028. 
